# Utility of High-Sensitivity and Conventional Troponin in Patients Undergoing Transcatheter Aortic Valve Replacement: Incremental Prognostic Value to B-type Natriuretic Peptide

**DOI:** 10.1038/s41598-019-51371-x

**Published:** 2019-10-17

**Authors:** Yukari Kobayashi, Juyong B. Kim, Kegan J. Moneghetti, Michael Fischbein, Anson Lee, Claire A. Watkins, Alan C. Yeung, David Liang, Mehmet O. Ozen, Utkan Demirci, Raffick Bowen, William F. Fearon, Francois Haddad

**Affiliations:** 1 Division of Cardiovascular Medicine, Stanford University School of Medicine, Stanford, CA United States; 2 Stanford Cardiovascular Institute, Stanford, CA United States; 3 Division of Cardiothoracic Surgery, Stanford University School of Medicine, Stanford, CA United States; 4 Bio‐Acoustic‐MEMS in Medicine (BAMM) Laboratories, Canary Center at Stanford for Cancer Early Detection, Department of Radiology, Stanford University School of Medicine, Palo Alto, CA United States; 5 Department of Pathology, Stanford University School of Medicine, Stanford, CA United States

**Keywords:** Predictive markers, Prognostic markers, Disease-free survival

## Abstract

High-sensitivity Troponin (hs-Tn) has emerged as a useful marker for patients with myocardial injury or heart failure. However, few studies have compared intermediate and hs-Tn in patients undergoing transcatheter aortic valve replacement (TAVR). Moreover, there remains uncertainty of which thresholds are the most useful for discriminating ventricular dysfunction or outcome. In this study we prospectively enrolled 105 patients with severe aortic stenosis (AS) who underwent TAVR as well as blood sampling for high-sensitivity (hs-TnI) and conventional troponin I (EXL-LOCI and RXL) assessment. Patients underwent comprehensive pre-procedure echocardiography. Ventricular dysfunction was defined using left ventricular mass index (LVMI), LV global longitudinal strain (LVGLS) and LV end-diastolic pressure. The mean age was 84.0 ± 8.7 years old and 60% were male sex with mean transaortic pressure gradient of 50.1 ± 16.0 mmHg and AVA of 0.63 ± 0.19 cm^2^. When using a threshold of 6 ng/L, 77% had positive hs-TnI while 27% had positive hs-TnI using recommended thresholds (16 ng/L for female and 34 ng/L for male). Troponin levels were higher in the presence of abnormal LV phenotypes. The strongest correlate of troponin was LVMI. During median follow-up of 375 days, 21 patients (20%) died. Lower threshold of hs-TnI and EXL-TnI was more discriminatory for overall mortality (Log-rank P = 0.03 for both), while higher threshold of hs-TnI (p = 0.75) and RXL-TnI were not (p = 0.30). Combining hs-TnI and BNP improved to predict long-term outcome (p = 0.004). In conclusion, hs-TnI levels correlated with the degree of LV dysfunction phenotypes. Furthermore, applying a lower threshold for hs-TnI performed better for outcome prediction than a recommended threshold in patients undergoing TAVR. Combining hs-TnI with BNP helped better risk stratification.

## Introduction

Cardiac troponin (Tn) is one of the cardiac markers which is recommended to be used for the diagnosis and management of acute coronary syndrome (ACS)^1^. It has been emerged as useful not only for the diagnosis of myocardial injury but also in patients with heart failure or pressure overloaded ventricles^2,3^. Chin *et al*. have reported that plasma Tn concentrations are associated with LV hypertrophy and myocardial fibrosis in patients with aortic stenosis (AS)^4^. Furthermore, several studies have demonstrated that the myocardial injury after transcatheter aortic valve replacement (TAVR) was common when assessed by the increase of Tn after the procedure, however, the increment was not associated with outcome^5–7^.

Tn can be assessed using assays of different sensitivity. Intermediate-sensitivity (is) Tn or low-sensitivity (ls) Tn has been used conventionally for a diagnosis of ACS. Recent advances in technology have allowed more sensitive and precise assays of circulating Tn levels (high-sensitivity (hs) Tn), leading better identification of higher risk patients with the negative is- or ls-Tn^2,3^. To date, few studies have compared the performance of hs- or conventional Tn in patients with severe AS undergoing TAVR. Moreover, there remains controversy of best threshold to use in different conditions. B-type natriuretic peptide (BNP) is another important cardiac biomarker, which is released from ventricle due to pressure and/or volume overload, and has been reported to have important prognostic value in multiple cardiovascular diseases^8^. However, few studies have assessed the compremental value of these parameters on outcome prediction in patients with AS undergoing TAVR.

Therefore, in this study we first sought to determine the prevalence of cardiac dysfunction phenotypes according to different thresholds and different assays. We then sought to determine correlates of elevated Tn levels. Finally, we sought to explore the relationship between Tn levels and the outcome in patients following TAVR and investigate additional prognostic value of hs-TnI to BNP.

## Methods

### Study population

Adult patients with severe AS who received TAVR at Stanford University Medical Center and underwent the complete set of troponin assessments, including hs-TnI, conventionally used TnI were prospectively included in this study. Patients with prior valve replacement surgery, known active infection or cancer, on immunosuppressive therapy, or end-stage renal disease on dialysis were excluded. Furthermore, 150 healthy controls who had blood testing for hs-TnI from our Stanford healthy aging cardiovascular institute database were selected to derive the threshold for hs-TnI. This study was approved by the Stanford Institutional Review Board with all protocols conducted in accordance with relevant guidelines and regulations. Informed consent was obtained from all patients enrolled.

### Biomarkers analysis

Blood sampling was performed after anesthesia had been administered but before the aortic valve was treated. Hs-TnI and BNP were measured using Abbott ARCHITECT instrument, which was performed at Abbott Core Laboratory. The total precision defined by the % coefficient of variation (CV) was 5% and the limit of quantitation which corresponds to the TnI concentration at which CV = 10% was reported to be 3 ng/L^9^. Conventional TnI was measured on a Dimension EXL^TM^ regarded as intermediate-sensitivity and RXL^TM^ (Siemens Health Care) regarded as low-sensitivity, which was performed in the chemistry section of the core clinical laboratory at Stanford. For EXL^TM^ (EXL-TnI), the within-run imprecision defined by the %CV and obtained at each Bio-Rad quality control level was 2.2%, 1.2% and 2.2%, while the between-run imprecision was 3.1%, 2.9% and 2.5%. The assay total imprecision was 4.1%, 3.2% and 3.3% for each control level. The limit of quantitation which corresponds to the TnI concentration at which CV = 10% was 0.056 ng/mL. For RXL^TM^ (RXL-TnI), the assay total imprecision was 12.6% at 0.43 ng/mL and 9.3% at 1.80 ng/mL for low and high quality control specimens. The limit of quantitation which corresponds to the TnI concentration at which CV = 10% was 0.14 ng/mL and the lower detection limit was 0.04 ng/mL. TnI measured on Dimension EXL is guideline acceptable at a level 1, and the one measured on RXL is considered clinically usable at a level 1 at Stanford Health Care. All biomarkers were analyzed blinded to the clinical characteristics of the study.

### Echocardiography

Echocardiography was performed using commercially available echocardiographic systems (iE33, and EPIQ 7C; Philips Medical Imaging, Eindhoven, the Netherlands), according to the American Society of Echocardiography guideline recommendations^10^. Left ventricular (LV) end-systolic and end-diastolic volumes and ejection fraction (LVEF) were calculated with biplane Simpson’s method. Transmitral pulse Doppler velocities and tissue Doppler velocities of the mitral annulus were measured from apical 4-chamber view and the E/e’ ratio was obtained by averaging the septal and lateral values. LV longitudinal strain was measured using Lagrangian strain by manual tracing as previously described^11^. Briefly, we measured the myocardial initial length in end-diastole (L_0_) and final length in end-systole (L_1_) and calculated LV strain values as 100 × (L_1–_L_0_)/L_0_^12^. Global longitudinal strain (GLS) represents the average values of longitudinal strain calculated from the apical 4-, 3-, and 2-chamber views.

### Statistical analysis

Results are expressed as mean ± standard deviation for continuous variables or median and interquartile range when not normally distributed, or as the frequency and percentage for categorical variables. Comparison of groups was performed using Student’s t-test for continuous variables and Chi-square test or Fisher Exact Test as appropriate for categorical variables. Univariable regression analysis was performed to evaluate the association of parameters with the hs-TnI values and then the parameters were entered in multivariable stepwise analysis. The log-rank test was performed to compare the outcome and shown in Kaplan-Meier plots using the different cut-offs.

P values < 0.05 were considered statistically significant. Analyses were performed using SPSS version 21 (SPSS Inc, Chicago, Illinois).

## Results

Among patients enrolled, we included 105 patients in the analysis of this study who underwent the complete set of troponin assessments, including hs-TnI, conventional EXL-TnI and RXL-TnI. The mean age was 84.0 ± 8.7 years old and 60% were male sex (Table 1).

**Table 1:** Patients’ characteristics.

	N = 105
Age, years	84.0 ± 8.7
Male sex, n (%)	63 (60)
Heart rate, bpm	72 ± 14
Systolic blood pressure, mmHg	125 ± 17
Diastolic blood pressure, mmHg	69 ± 11
Diabetes mellitus, n (%)	33 (31)
History of coronary artery disease, n (%)	57 (54)
Hs-TnI, ng/L	13.3 [6.45–29.1]
EXL-TnI, ng/mL	0.021 [0.0095–0.047]
RXL-TnI, ng/mL	0.000 [0.000–0.02]
BNP, ng/L	276.2 [142.3–598.6]
Ln BNP, ng/L	5.70 ± 1.02
**Echocardiography**	
Interventricular septum, cm	1.23 ± 0.22
Posterior wall, cm	1.19 ± 0.22
LV dimension, cm	4.7 ± 0.9
Aortic valve area, cm^2^	0.63 ± 0.19
Mean transaortic pressure gradient, mmHg	50.1 ± 16.0
Peak transaortic pressure gradient, mmHg	83.4 ± 27.0
LVEF, %	54.1 ± 12.5
LVGLS, %	−13.0 ± 3.2
LVEDV, ml	108.5 ± 40.2
LVESV, ml	53.2 ± 33.6

BNP, brain natriuretic peptide; EDV, end-diastolic volume; ESV, end-systolic volume; GLS, global longitudinal strain; hs, high-sensitivity; is, intermediate-sensitivity; ls, low-sensitivity; LV, left ventricular; EF, left ventricular ejection fraction; Tn, troponin.

**Figure 1:**
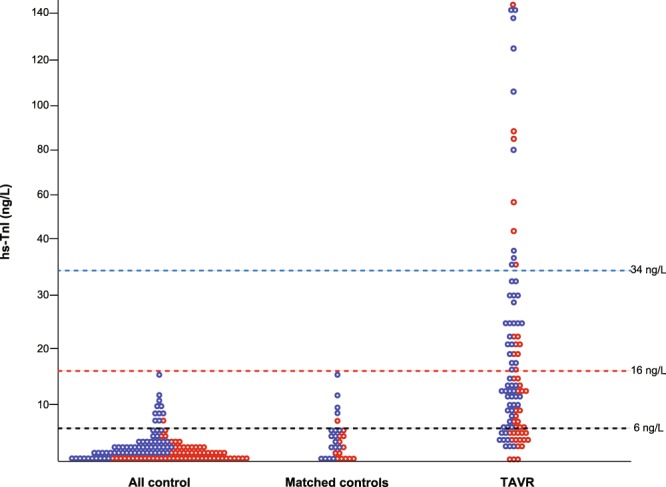
Distribution of hs-TnI in all healthy, age-matched healthy, and patients with TAVR groups. The red represents female sex and the blue represents male.

The control group consisted of 150 subjects (77 male and 73 female) with a mean age of 60.6 ± 16.6 (from 20 to 93) years old. The median value of hs-TnI was 1.15 [0.2–2.7] ng/L and upper 95th percentile limit was calculated at 8.44 ng/L. When healthy controls were selected who were matched for age and sex to the TAVR group (N = 30), the median value of hs-TnI was 2.95 [0.2–5.4] ng/L and upper 95th percentile limit was calculated at 13.47 ng/L. In the TAVR group, the median value of TnI was 13.3 [6.5–28.6] ng/L. The distribution of TnI for each cohort is shown in Fig. 1. In terms of BNP, the median value was 276.2 ng/L [142.3–598.6] (Table 1). Among patients, 47 patients (44.8%) presented with positive for both hs-TnI (using the threshold as 6 ng/L) and BNP (using the threshold as 250 ng/L^13^), 43 patients (41.0%) presented with either of them, and 15 patients (14.3%) presented with negative for both of them.

**Figure 2:**
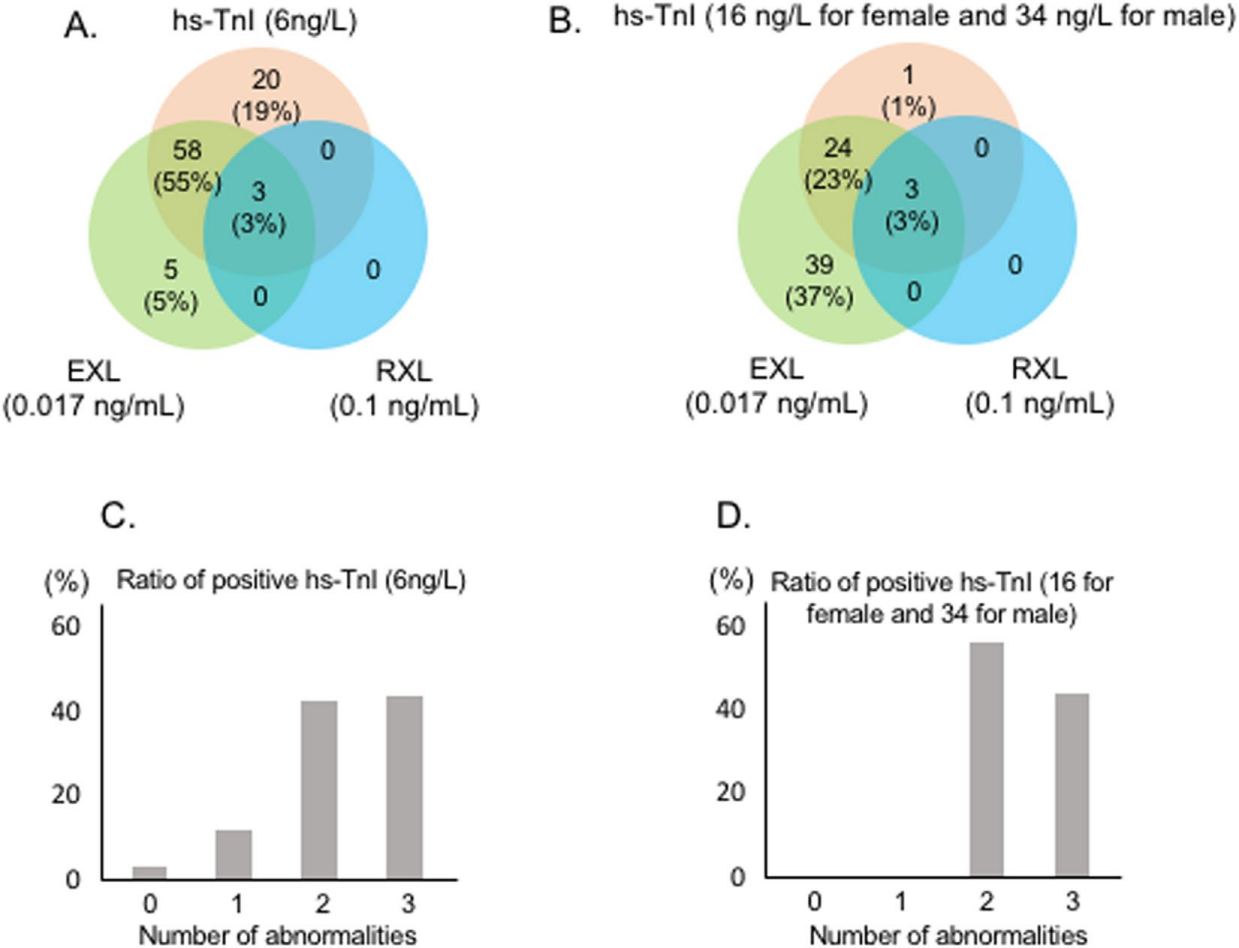
The prevalence of positive TnI in each assay and different thresholds and the ratio of positive-TnI according to the number of abnormalities. The panels (A,B) represent Venn diagrams demonstrating the overlap between positive TnI in hs-TnI, EXL-TnI, and RXL-TnI according to the different thresholds of hs-TnI as 6 ng/L (A) and 16 ng/L for male and 34 ng/L for male (**B**). The panels (C,D) represent the ratio of positive hs-TnI according to the number in the features of maladaptation using different threshold of hs-TnI of 6 ng/L (**C**) and 16 ng/L for male and 34 ng/L for male (**D**). LV, left ventricular; TnI, troponin I.

We selected the different thresholds of hs-TnI according to the previously used value (6 ng/L from upper quintile of BiomarCaRE population^14^, lower threshold), or currently recommended value determined by the manufacturer (34 ng/L for male and 16 ng/L for female^9^, strict threshold). Figure 2A,B present the venn diagrams demonstrating the overlap between the number of patients with positive result in the three TnI assessments according to the different hs-TnI thresholds. All three patients with positive RXL-TnI also had positive EXL-TnI and hs-TnI. Among patients with negative hs-TnI, 5 out of 24 patients (21%) presented positive in EXL-TnI when using a threshold of 6 ng/L, while 39 out of 77 patients (51%) presented negative hs-TnI and positive EXL-TnI when using strict threshold. Among controls, 11 subjects (7.3%) presented positive hs-TnI using the threshold as 6 ng/L, while no subjects presented positive using the strict threshold (Fig. 1).

### Phenotype comparison

Among 105 patients, 85 patients (81%) presented with impaired GLS (threshold as −16%), 55 patients (52%) presented LV hypertrophy (threshold as 95 g/m^2^ for female and 110 g/m^2^ for male), and 87 patients (85%) presented elevated LV filling pressure defined as LV end-diastolic pressure >12 mmHg. As shown in Table 2, worse LVEF, LVGLS, and increased LVMI were related to increased hs-TnI, and among those parameters only increased LVMI was retained as a parameter related to increased TnI in multivariable analysis (R = 0.26, beta = 0.26, p = 0.009). In terms of the relationship of cardiac function with EXL-TnI, only increased LVMI was related to increased EXL-TnI (R = 0.21, beta = 0.21, p = 0.03).

**Table 2:** Parameters related to hs-TnI.

Variables	Univariable			Multivariable		
	Beta	B	P value	Beta	B	P value
Age	0.14	0.66	0.16			
Male sex	0.16	13.09	0.11			
History of coronary artery disease	0.017	1.35	0.87			
Diabetes mellitus	0.004	0.36	0.97			
eGFR	0.10	0.18	0.32			
LV mass index	0.26	0.23	0.009	0.27	0.23	0.008
LVEF	−0.21	−0.67	0.04			
LVGLS (absolute)	−0.20	−2.55	0.04			
Mass to volume ratio	0.19	4.75	0.053			
Average E/e′	0.003	0.001	0.96			
LVEDP	−0.06	−0.26	0.56			

EDP, end-diastolic pressure; eGFR, estimated glomerular filtration rate; GLS, global longitudinal strain; hs, high-sensitivity; is, intermediate-sensitivity; ls, low-sensitivity; LV, left ventricular; LVEF, left ventricular ejection fraction.

Figure 2C,D show the ratio of patients who presented positive hs-TnI according to the number of abnormalities assessed by worsened LVGLS, increased LVMI, and increased LV end-diastolic pressure. When using the lower threshold of 6 ng/L as cut-off, the ratio of patients with positive hs-TnI increased with the number of abnormalities (Fig. 2C). When using the strict criteria, no patients presented positive in patients with none or one abnormality (Fig. 2D).

### Exploratory outcome analysis

**Figure 3:**
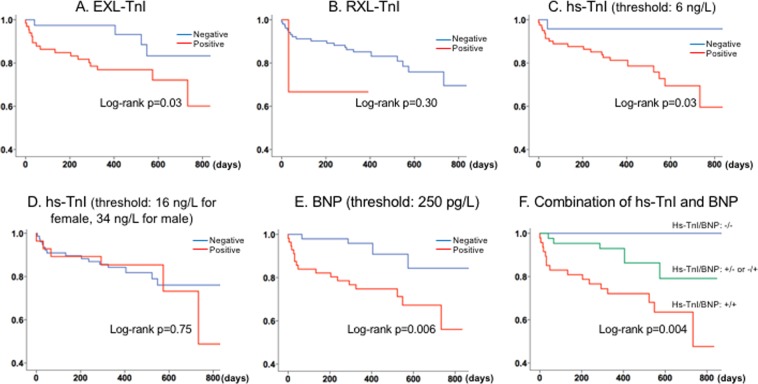
Kaplan-Meier curves of all-cause death. EXL-TnI differentiated the outcome (**A**) but not RXL-TnI (**B**). Hs-TnI differentiated the outcome when the threshold of 6 ng/L was used (**C**), however, it did not when the threshold of 16 ng/L for female and 34 ng/L for male was used (**D**). When combined hs-TnI and BNP, patients with both negative had the best outcome. while those with both positive had the worst outcome (**F**). BNP, B-type natriuretic peptide; LV, left ventricular; TnI, troponin I.

During a median follow-up of 374 [339, 640] days, 21 patients (20%) died among whom one patient died related to procedure on the same day. Kaplan-Meier curves analysis found that EXL-TnI differentiated the outcome (log-rank p = 0.003) (Fig. 3A) but not RXL-TnI (Log-rank p = 0.30) (Fig. 3B) among the conventional TnI. Hs-TnI differentiated the outcome when the lower threshold of 6 ng/L was used (Fig. 3C), however, it did not when the strict threshold was used (Fig. 3D). As shown in Fig. 3E, BNP also differentiated the outcome (Log-rank p = 0.006). When combined with hs-TnI and BNP, patients with both negative had the best outcome while patients with both positive had the worst outcome (Log-rank p = 0.004) (Fig. 3F).

## Discussion

The main finding of our study is that myocardial injury assessed by hs-TnI or EXL-TnI is common in patients with AS undergoing TAVR. Hs-TnI was related best to the LVMI and its elevation was closely related with abnormal LV phenotypes. Finally, the lower threshold of hs-TnI may be more useful for outcome prediction than higher thresholds, furthermore, the combination of hs-TnI with BNP improved the outcome prediction in those population.

Cardiac Tn determination is one of the standards of practice for the diagnosis of acute myocardial infarction. EXL- or RXL-Tn has been conventionally used, however, the use of high-sensitivity assays is actively studied for performance in early rule out^15–17^. Hs-Tn levels have been further demonstrated to correlate with the prevalence of cardiovascular risk factors, cardiac hypertrophy or dysfunction^18^. For example, Chin *et al*. investigated 122 patients with AS using cardiac magnetic resonance and echocardiography and found that LVMI and LV fibrosis were independently associated with hs-TnI concentrations^4^. In the study, they also followed up another group of 131 patients with AS for median of 10.6 years and reported that hs-TnI concentration was associated with an increased risk of surgical aortic valve replacement or cardiovascular death. In patients following TAVR, some studies have demonstrated that hs-TnI was commonly elevated after the procedure^5–7^, while the degree of hs-TnI elevation was not associated with outcome. In those studies, they did not assess the impact of hs-TnI before the procedure on outcome.

The assay used as a threshold of hs-TnI using the Abbott Architect platform is well validated for a diagnosis of myocardial infarction as 16 ng/L for women and 34 ng/L for men^19^. This definition has the advantage to consider gender difference as well as to keep high specificity. On the other hand, lower thresholds are often advocated in patients with different conditions such as heart failure or pressure overload states. Recently, Adamson *et al*. measured hs-TnI by Abbott ARCHITECT_*STAT*_ TnI assay in 1599 patients with chronic obstructive pulmonary disease and compared the outcome between the patients with TnI < 2.3 ng/L and those with TnI ≥ 7.7 ng/L, which are the lowest and highest quintile regardless of gender difference^20^. They found the relationship between hs-TnI and cardiovascular events in the population even though those values are within normal range in the validated threshold.

The threshold of 6 ng/L is within normal range. However, the lower value differentiated the cardiac function abnormality including systolic and diastolic dysfunction as well as abnormal morphology. In addition, this value well predicted the outcome following TAVR. This differentiating ability seems similar to EXL-TnI. On the other hand, the strict threshold did not differentiate the outcome, suggesting that greater specificity may lose some predictive power, being too high for outcome prediction.

Another important finding of our study is the impact of combining the information of BNP and hs-TnI on predicting outcome in patients with AS following TAVR. BNP is a hormone produced from the heart in response to the pressure or volume overload and known to be valuable to predict outcome in multiple cardiovascular diseases^8^. Some studies have investigated the usefulness of BNP for outcome prediction in patients with AS undergoing TAVR^21,22^. Consistent with those studies, patients with higher BNP had worse outcome in our study. Furthermore, our study demonstrated that combining the information of hs-TnI and BNP helped better differentiate the outcome in patients with AS undergoing TAVR. This suggests that even either of them presented negative, the additional information of the other parameters would help better risk stratification before the procedure.

This study also validates previously findings that an increased hs-TnI is related to cardiac function impairment^4^. This has two implications in the field. First, the fact that LVMI was independently related to hs-TnI, raises the possibility of using this phenotype to differentiate those patients at higher risk. Secondly, future studies should consider the importance of preventing progression of LV hypertrophy to improve procedural or overall outcome in TAVR.

There are some limitations in this study. First, the number of healthy controls was relatively small and we did not derive the threshold depending on gender differences from our healthy cohort. However, to differentiate the threshold between male and female was not help detect disease or predict outcome in our population. In addition, the number of patient population was also small in our study, therefore, further larger studies are warranted to confirm our findings. We did not assess the post-procedure hs-TnI or the increase of hs-TnI after the procedure. However, our study explored that the pre-procedural hs-TnI predicted outcome and it would be more helpful stratify the risk before the procedure.

## Conclusion

Applying a lower threshold helped detect higher incidence of cardiac dysfunction phenotypes, in addition, lower threshold may be more useful for outcome prediction than higher thresholds in patients with AS undergoing TAVR. Hs-TnI was related best to the LVMI and its elevation was closely related with abnormal LV phenotypes. Finally, combining hs-TnI with BNP would help better differentiate the outcome in this population.

## Data Availability

The datasets generated during and/or analyzed during the current study are available from the corresponding author on reasonable request.
